# Reply to “Pathway for ascertaining the role of uric acid in neurodegenerative diseases,” Roman Youssef

**DOI:** 10.1002/dad2.12328

**Published:** 2022-06-21

**Authors:** Mats Dehlin, Tahzeeb Fatima, Lennart T.H. Jacobsson, Silke Kern, Anna Zettergren, Kaj Blennow, Henrik Zetterberg, Lena Johansson, Ingmar Skoog

**Affiliations:** ^1^ Department of Rheumatology and Inflammation Research Sahlgrenska Academy University of Gothenburg Gothenburg Sweden; ^2^ Department of Clinical Sciences Lund Section of Rheumatology Lund University Lund Sweden; ^3^ Department of Psychiatry and Neurochemistry at Institute of Neuroscience and Physiology Sahlgrenska Academy University of Gothenburg Gothenburg Sweden; ^4^ Clinical Neurochemistry Laboratory Sahlgrenska University Hospital Mölndal Sweden; ^5^ UK Dementia Research Institute at UCL London UK; ^6^ Department of Neurodegenerative Disease UCL Institute of Neurology London UK

We have read with interest the reply by Dr. Roman Youssef to our article “Association between serum urate and CSF markers of Alzheimer's disease pathology in a population‐based sample of 70‐year‐olds” published in this journal in December 2021.

In the study we found an association between serum urate and the cerebrospinal fluid (CSF) biomarker amyloid beta (Aβ)_42_ in cognitively unimpaired 70‐year‐old men, suggesting a protective effect of urate against deposition of amyloid in the brain. In his letter, Dr. Youssef suggests that our study could have benefited from adjustments for factors known to be associated to increased levels of serum urate, hyperuricemia, such as renal function and body mass index (BMI).

However, in our study we focused on the cross‐sectional relation between serum urate and CSF biomarkers; that makes the explanatory factors behind the current urate levels less important, and we, therefore, saw no reason to adjust for them. On the other hand, renal function may be a risk factor for Alzheimer's dementia (AD)^1^ and to complicate matters BMI has a dual relation with AD risk, the so‐called obesity paradox.^2^

Dr. Youssef raises the question on the relation between levels of urate in serum compared to CSF and the potential role of the blood‐brain barrier in this. This is an important question, and future studies could benefit from measurement of urate in CSF, although published data seem to support a strong relation between urate in the two compartments.^3^, ^4^

The possible protective effect of urate against Aβ_42_ deposition needs to be studied in other settings and in longitudinal analysis, and if confirmed may pose as a modifiable risk factor that may alter the course of AD.

## CONFLICTS OF INTEREST

Tahzeeb Fatima, Lennart T.H. Jacobsson, Anna Zettergren, Mats Dehlin, and Ingmar Skoog declare no conflicts of interest. Silke Kern has served as a consultant, on advisory boards for Geras Solutions, unrelated to the results presented in this paper. Kaj Blennow has served as a consultant, on advisory boards, or on data‐monitoring committees for Abcam, Axon, Biogen, JOMDD/Shimadzu, Julius Clinical, Lilly, MagQu, Novartis, Roche Diagnostics, and Siemens Healthineers, and is a cofounder of Brain Biomarker Solutions in Gothenburg AB (BBS), which is a part of the GU Ventures Incubator Program, all unrelated to the results presented in this paper. Henrik Zetterberg has served on scientific advisory boards for Denali, Roche Diagnostics, Wave, Samumed, SiemensHealthineers, Pinteon Therapeutics, Nervgen, and CogRx; has given lectures in symposia sponsored by Fujirebio, Alzecure, and Biogen; and is a co‐founder of Brain Biomarker Solutions in Gothenburg AB (BBS), which is a part of the GU Ventures Incubator Program, all unrelated to the results presented in this paper.
